# Structural Health Monitoring: An IoT Sensor System for Structural Damage Indicator Evaluation

**DOI:** 10.3390/s20174908

**Published:** 2020-08-31

**Authors:** Mirco Muttillo, Vincenzo Stornelli, Rocco Alaggio, Romina Paolucci, Luca Di Battista, Tullio de Rubeis, Giuseppe Ferri

**Affiliations:** 1 Department of Industrial and Information Engineering and Economics (DIIIE), University of L’Aquila, Piazzale Pontieri 1, Monteluco di Roio, 67100 L’Aquila, Italy; mirco.muttillo@graduate.univaq.it (M.M.); romina.paolucci@graduate.univaq.it (R.P.); tullio.derubeis@univaq.it (T.d.R.); giuseppe.ferri@univaq.it (G.F.); 2 Department of Civil, Construction-Architectural and Environmental Engineering (DICEAA), University of L’Aquila, Via Giovanni Gronchi 18, Zona industrial di Pile, 67100 L’Aquila, Italy; rocco.alaggio@univaq.it; 3 Department of Information Engineering, Computer Science and Mathematics (DISIM), University of L’Aquila, Via Vetoio, Coppito, 67100 L’Aquila, Italy; luca.dibattista1@graduate.univaq.it

**Keywords:** structural health monitoring, IoT structural monitoring, damage indicator, damage detection system

## Abstract

In the last decades, the applications of structural monitoring are moving toward the field of civil engineering and infrastructures. Nevertheless, if the structures have damages, it does not mean that they have a complete loss of functionality, but rather that the system is no longer in an optimal condition so that, if the damage increases, the structure can collapse. Structural Health Monitoring (SHM), a process for the identification of damage, periodically collects data from suitable sensors that allow to characterize the damage and establishes the health status of the structure. Therefore, this monitoring will provide information on the structure condition, mostly about its integrity, in a short time, and, for infrastructures and civil structures, it is necessary to assess performance and health status. The aim of this work is to design an Internet of Things (IoT) system for Structural Health Monitoring to find possible damages and to see how the structure behaves over time. For this purpose, a customized datalogger and nodes have been designed. The datalogger is able to acquire the data coming from the nodes through RS485 communication and synchronize acquisitions. Furthermore, it has an internal memory to allow for the post-processing of the collected data. The nodes are composed of a digital triaxial accelerometer, a general-purpose microcontroller, and an external memory for storage measures. The microcontroller communicates with an accelerometer, acquires values, and then saves them in the memory. The system has been characterized and the damage indicator has been evaluated on a testing structure. Experimental results show that the estimated damage indicator increases when the structure is perturbed. In the present work, the damage indicator increased by a maximum value of 24.65 when the structure is perturbed by a 2.5 mm engraving.

## 1. Introduction

The monitoring applications cover various disciplines, from aerospace to the diagnostics of malfunctions of machines and mechanical systems, in the last years have been utilized in civil engineering and infrastructure. This topic is also studied as scientific research, as evidenced by a large number of articles in the related literature. The purpose of monitoring is to know the behavior of a building in a timely manner and from different points of view. For this reason, energy monitoring systems [1,2,3,4,5,6,7] are often used combined with environmental monitoring systems and sensors [8,9,10,11,12,13,14,15]. This combination allows the building to remain in optimal conditions in terms of consumption and durability over time and for its increasing connection to the Internet of Thing (IoT) world.

On the other hand, the development of an optimal monitoring system is still an open challenge [16]: determining which of the existing ones is the most appropriate is absolutely not trivial. Indeed, the structures themselves are incredibly heterogeneous, both in terms of construction technology and in terms of age. Therefore, it is challenging to find a system that fits them all indiscriminately. Furthermore, monitoring is an extremely multidisciplinary topic and it is extremely complex to take into account all the variables involved.

Structural monitoring systems encounter two types of challenges: aging, with consequent and gradual loss of operating conditions, and the occurrence of a sudden and unexpected event, as an earthquake [17]. However, there is a certain heterogeneity in the methods of applying structural monitoring. The objectives go towards a more precise detection capacity, easier management, and storage of data (even when they are in large quantities), timeliness, and reliability of the information provided [18].

The application of a system that allows structural monitoring has a specific name in the literature, which is Structural Health Monitoring (SHM). Schubel et al. [19] presented a review of structural health monitoring techniques for wind turbine blades. Indeed, the potential of the structural monitoring for these specific application savings to manufacturing time and reduces the cost of the quality control phases. Furthermore, the complete knowledge of the behavior of the structure through monitoring permits better design and manufacturing. An essential other application of structural health monitoring is for aircraft. Diamanti et al. [20] presented an SHM technique for composite structures of the aircraft. The size of the critical damage has been determined by the defect of the composite structures that can be found with a visual inspection, electromagnetic testing, ultrasonic inspection, and other methods. Furthermore, fiber optic sensor technology is increasingly used for aircraft monitoring to reduce the cost of maintenance and to identify damage in the structure [21].

The structural monitoring, in addition to ensuring that the structure is always in excellent health, also exempts from the need to carry out visual inspections and substitute the use of more conventional methods (for example ultrasound methods). The advantage of the SHM is characterized by being a very promising alternative and efficient with respect to the conventional methods. Indeed, visual inspections are not always possible and are, in any case, expensive in terms of time and money, while the use of conventional methods cannot give up on the operator experience [22].

Therefore, SHM is generally characterized by a non-destructive approach allowing continuous and autonomous monitoring thanks to the use of integrated sensors [23,24]. A typical structural monitoring system, then, is made up of a sensor system, a data processing system, and a health evaluation system [25].

There is a wide variety of monitoring solutions, in addition to the number and complexity of sensors. Indeed, there are methods based on the study of natural frequency [26], which allows the study of vibrations. Furthermore, there are methods widely used in the case of sudden structural degradations [27]. Other methods are based on the study of modal forms [28], where a system is capable of limiting false alarms. Then there are the so-called “hybrid” methods because they are based on the study of approaches [29]. Methods based on the use of artificial neural networks, capable of learning from past data and formulating predictions on future evolutions of the structure, are also an object of study [18,30].

Concerning the used methodology, the heart of SHM is damage detection. The occurrence of damage, in fact, can entail, and often does, changes in the characteristics of the structure (for example with regard to stiffness) [31] which, properly detected, needs maintenance work to avoid the aggravation of the situation since, in the long run, the structure itself collapses. It follows that the rapid identification of the damage is a fundamental step in SHM. A fully developed system should be capable of detecting and evidencing in near real-time the occurrence of a structural anomaly, identifying its location, and associating it with a type of structural damage and intensity [32]. There are two ways to monitor a structure. When its global behavior is analyzed, and the structure is considered as a single system, we speak of global damage identification. On the other hand, when we focus only on certain elements considered critical or already weakly damaged we speak of local identification of the damage [33].

In this work, an enhanced version with a different use of the proposal shown in [34] is presented. The proposed monitoring system for structural health is based on a microcontroller and two triaxial accelerometric sensors. The data returned, and subsequently suitably processed, allows to determine the identification of the damage indicator on an engrave steel bar.

## 2. Literature Background of Structural Health Monitoring

Starting from this common concept, there were different ways in which SHM has declined over the years, also in reference to different degrees of complexity. In [35], for example, an SHM system was born practically by chance because the rather poor sensors installed at the Meazza Stadium in Milan, were not originally intended to monitor the structure. Only later, it became clear that the information returned was also interesting from that point of view. In [36], on the other hand, a much more complex system is presented, which makes use of more than 600 sensors, testifying how wide and varied this field of research is.

Wang et al. [37], proposed a wireless structural health monitoring system for real-time data acquisition. This kind of system is limited in the number of sensors and the capability of the synchronization of the samples. Indeed, to increase the sampling rate, the number of sensors connected in the same network decreases. Therefore, with a sampling frequency of 100 Hz, the number of nodes is equal to 12. Furthermore, to use a wireless monitoring system in a large structure, such as a bridge, a peer-to-peer wireless sensor network must be designed and improved. Hu et al. [38] developed a wireless monitoring system integrated into the Zhengdian Highway Bridge for structural health monitoring. This system is able to acquire samples in continuous mode using a microcontroller and ADC to acquire analog accelerometers. The main problem of analog accelerometers is the output drift due to the temperature, and compensation circuits are needed. The work shows the limitation of the proposed wireless system in terms of output data. Indeed, the results are limited due to the noise interference of the analog circuits and data losses of the transmission.

The main component of the proposed monitoring system is the accelerometer ADXL355 [39]. This accelerometer is a digital sensor that is able to acquire the three-axis accelerations internally and send them to an external microcontroller. A better explanation of the proposed monitoring system is given in Section 3.1, and before presenting the proposed IoT sensor system for structural health monitoring, a literature review of the related works that used the same accelerometers is proposed.

Multiple works in the literature [40,41,42,43,44,45,46,47] present a monitoring system for structural health monitoring using the accelerometer ADXL355. These works are divided into the system based on a wireless sensor network (WSN) and wired monitoring systems. Valenti et al. [40] proposed a low-cost WSN for SHM and the system has been used for identification of modal frequency. The problem of this system is the synchronization of the samples. Indeed, the only synchronization refers to the start time and stop time that the master sends to the node. Other work that used a WSN was proposed by Wondra et al. [45]. The WSN was also used for monitoring the wind turbine tower to wind excitation. The limit of this system is the maximum sampling rates and the maximum nodes (31 Hz) and three nodes, respectively. Furthermore, synchronization is also a critical problem for this WSN.

The wired sensor monitoring system is an alternative to a WSN. An application for a wired system that used the ADXL355 is for earthquake detection [41,42,46]. Microseismic events is an important research field, and this kind of system can send warning messages when an event occurs. The limit of these systems is the small number of sensors that can be used. Indeed, the system is composed in general of one node that sends data to a web server. Other nodes are disconnected from each other and positioned at distances of kilometers. Pierleoni et al. [43] proposed a wired monitoring system with 64 samples per second without synchronization from each node that communicates the data through an ethernet connection. This system can appreciate the lowest modal frequency of the structures but not the highest due to the low sampling frequency. Quqa et al. [44], instead, realized a single node wired monitoring system for structural health. The system is able to identify the natural frequency and modal parameters in real-time. The system is based on a single-board computer and accelerometer ADXL355 that limits the synchronization and the maximum number of nodes connected in the same network. Navabian et al. [47] proposed an event monitoring system for structural health. This system acquires data if the event exceeds the threshold, and the duration of the acquisition is about 70 s. Although, like [40], the synchronization is also available from the start and stop acquire campaign.

Based on the literature review of the monitoring system that used the same accelerometer of the system proposed in this work, a summary of the comparison between wired and wireless can be done. The existing wired monitoring system has a very high cost, a typically low number of sensors that can be connected in the same network, high bandwidth, high sensor data rate, and very high sensor synchronicity. On the other side, the wireless monitoring system has a low cost, a high number of sensors that can be connected in the same network, limited bandwidth, low sensor data rate, and critical synchronization of nodes [48].

The main novelty of the wired proposed monitoring system, based on the previous analysis, are the following:

1. The high number of nodes that can be connected in the same network, the only limitation is due to the RS485 protocol;
2. High bandwidth;
3. High data rate;
4. High synchronization between nodes;
5. Low-cost system.

## 3. Materials and Methods

In the present work, for the detection of the damage on the beam model, we proceeded “by comparison”: first, some measurements were performed on the intact test structure, assuming this as the reference state; subsequently, they were repeated on the same structure deliberately perturbed through an incision of 2.5 mm.

**Figure 1:**
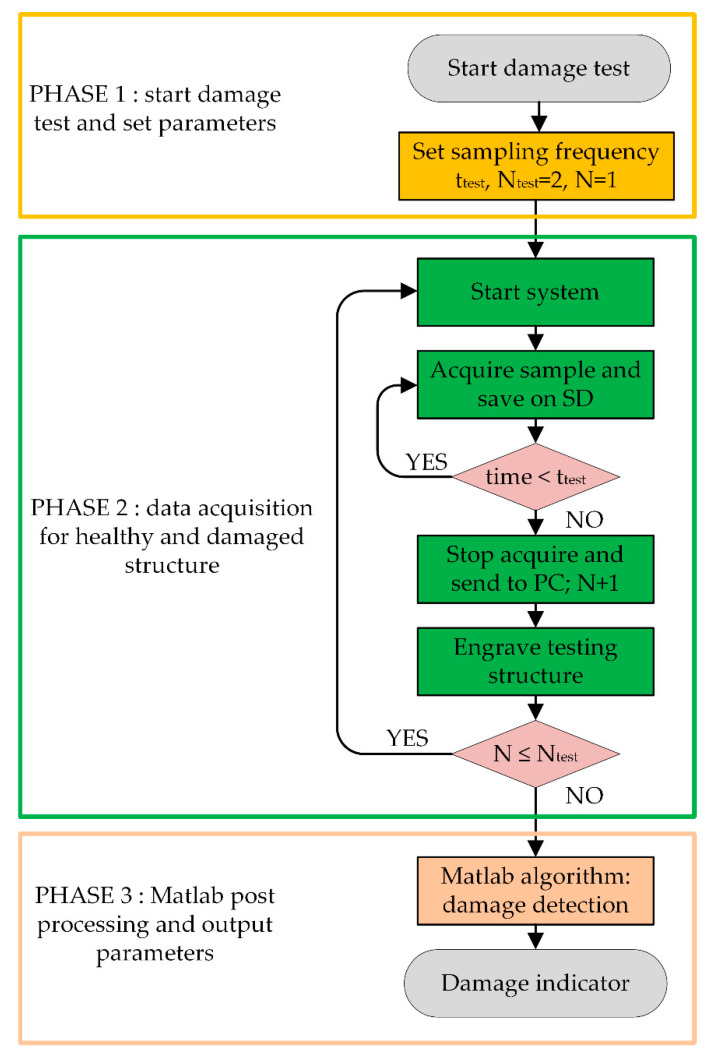
Damage indicator test operating phases.

The conducted test has been divided into three phases: in the first phase, the sampling frequencies and the duration of the test were chosen; in the second one, we proceeded to start the system, acquire the samples, save them in an SD card, stop the acquisition, and send the data to the PC; the third and final phase consisted of the post-processing of data through Matlab. The test operating phases to derive the damage indicator are shown in Figure 1 as a sort of flow-chart. Phase 2 consists of two tests: the first is the test with a healthy structure and the second is with a damaged structure. At system startup, N = 1. Therefore, the system acquires and saves to the SD card repeatedly for the test time. After that, the acquisitions were stopped, and data was sent to the PC. Instead, the second test consists of N = 2 and with the damaged structure. The system started and acquired the samples, saved them on the SD card, and then sent the data to the PC after the test time. The final phase is the post-processing of data for damage indication.

### 3.1. Damage Indicator

Damage detection is a problem that has been studied using various methods [49,50,51,52,53,54,55,56,57,58,59,60,61,62,63]. A fuzzy neural network for two-stage damage detection is presented by Jiang et al. [49]. A damage assessment based on a fuzzy neural network for the first stage has been performed. Whereas in the second stage, thanks to the using of the union of data fusion and fuzzy models, a final evaluation has been achieved. This approach can identify more patterns than the single-stage fuzzy model. Gui et al. [50] illustrated a three optimization algorithm for Gaussian kernel function parameters. These optimization algorithms are based on vector machines and are allowed to use them for damage detection. Other methods used the Particle Swarm Optimization algorithm [51], Operational Modal Analysis with dynamic measurements [52], frequency response functions with artificial neural network-based for damage detection [53], and 1D Convolutional Neural Networks for vibration-based damage detection and localization in real-time [54].

One of the first works on the identification of the damage index addressed from the one-dimensional point of view is that shown in [55], in which a method to evaluate the integrity of the structures non-destructively is shown. In particular, it is described how the measurement of vibrations carried out in a single station in the structure can be used, in combination with a suitable theoretical model, to indicate both the position and the extent of the damage.

The proposed experiment illustrates the application of the system for structural health monitoring using a damage detection method based on Stochastic Subspace Identification concepts [56]. The method, being based on a non-parametric test, does not require to explicitly know system parameters and is suitable for automatic data-driven damage detection monitoring of in-service structures.

Any damage diagnosis method requires the extraction of damage-sensitive features from the measurement data of the monitored system. The feature vector is generally defined in a way that it is approximately Gaussian distributed with zero mean in the reference (undamaged) state and non-zero mean in the damaged state, hence the designation of the residual vector [57,58]. Many residuals have been used in the literature [59,60]; in this paper, the subspace residual, representing the orthonormality defect between subspaces characterizing the dynamic response in the current state of the structure with respect to its reference, is adopted, specifically the robust subspace residual [61] less prone to changes in excitation covariance.

Measures of the dynamic response of the structure in its reference state are acquired over time to produce a statistical model of the residuals under changing environmental conditions [62]. If no structural damage occurs, the orthonormality assumption between the mentioned subspaces, evaluated for different data sets, remains approximately valid according to small residues. However, possible structural damage causes an increase in residues. This increase involves, with the choice of an adequate metric, a significant rise in the scalar damage indicator. Therefore, if this value falls beyond an appropriate threshold, it indicates the presence of damage [63].

### 3.2. System Description

**Figure 2:**
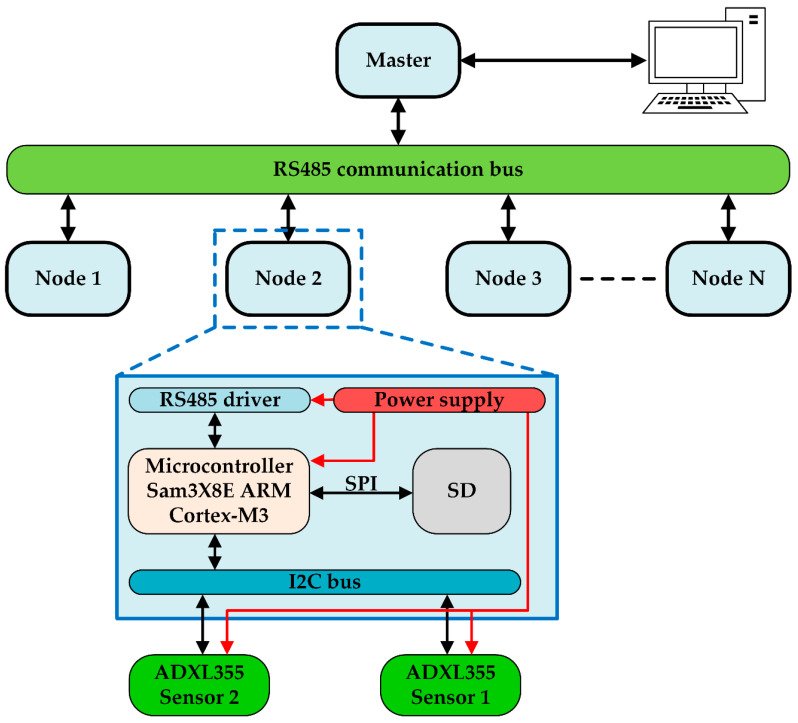
The scheme of the proposed monitoring system. In this scheme, the architecture of the node is presented.

The whole general scheme of the proposed monitoring system, with typical connection and node architecture, is shown in Figure 2.

The system is composed of nodes, described in more detail below, which, via the RS485 protocol, communicate with a master. The choice of this protocol is not casual: thanks to its characteristics, in fact, the nodes can be positioned even at a distance of hundreds of meters, without compromising their capability to communicate correctly with the master. This aspect is fundamental, as it allows the master to synchronize the various nodes, to recover the data sent by them and to forward them to the PC for post-processing via Matlab.

The single node, as seen in the previous figure, is made up, of a microcontroller, the SAM3X8E ARM Cortex-M3 [64], equipped with an integrated Direct Memory Access (DMA). One of its tasks is to manage communication with the master.

The microcontroller, of course, needs to be powered. However, since the total current consumption of the node is only 100 mA, it is configured as a low power system. This allows it to be powered also through photovoltaic panels with a battery and, then, the possibility of positioning the nodes even at great distances and in environments with no electricity.

In addition, the microcontroller provides data storage on an external SD (Secure Digital) card, whose presence is necessary considering that the number of samples acquired can quickly reach the order of millions. Therefore, at the end of the single acquisition, it is particularly useful to store the data on an SD card so that it can also be sent to the master later.

Another critical point of the system is related to the fact that the code execution time on the microcontroller for data acquisition and saving is much longer than the time occurring between one sample and the next. This always happens, even for sampling frequencies equal to 1 kHz, and makes it impossible to acquire and save all data sequentially. To avoid data loss, the integrated DMA has been used on the microcontroller, which, through direct access to memory, allows the bypassing of the control unit of the microcontroller itself and to store the data directly in the SD card memory.

To complete the description of the system, and in particular, of the nodes, it should be emphasized that, as can be seen in Figure 2, each of them is made up of two accelerometers, both connected to the same microcontroller. This is made possible by the fact that communication, in this case, is managed via the I2C protocol, which allows for connection of more than one device to the same bus, each with its own address, chosen via external hardware settings. In particular, the sensors used are integrated triaxial digital accelerometers. The fact that they are integrated makes it possible to calm the price of the system, making it effectively competitive even from a purely economic point of view. Specifically, the sensor model used is the Analog Device ADXL355, whose basic characteristics are voltage supply range equal to 2.25–3.6 V, settable range ±2, 4, 8 g for each axis, for ±2 g the sensitivity is 3.9 µg/LSB, low power device with 200 µA consumption in measurement mode and 20-bit internal analog-to-digital converter (ADC). The sensitivity of this sensor changes, according to the temperature, of ±0.01%/°C, with respect to the ambient value of 25 °C. The accelerometer has an internal temperature sensor that the microcontroller can read for the data compensation.

## 4. Experimental Set-Up

**Figure 3:**
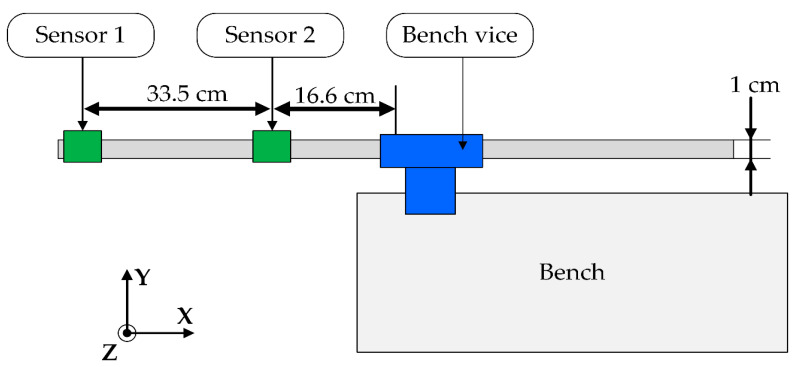
Testing the structure for the identification of the damage indicator.

The proposed monitoring system has been tested utilizing the experimental setup shown in Figure 3, where the identification of damage indicator procedure has been applied. The environment temperature test was equal to 25 °C, and under these test conditions, the sensitivity of the accelerometers does not change. The cantilever structure (aluminum bar) has been anchored with a bench vice. The two accelerometers of the acquisition node have been put on the aluminum bar. The first accelerometer has been mounted at the end of the bar and the second is positioned at 16.6 cm distance from the blocking point. 

**Figure 4:**
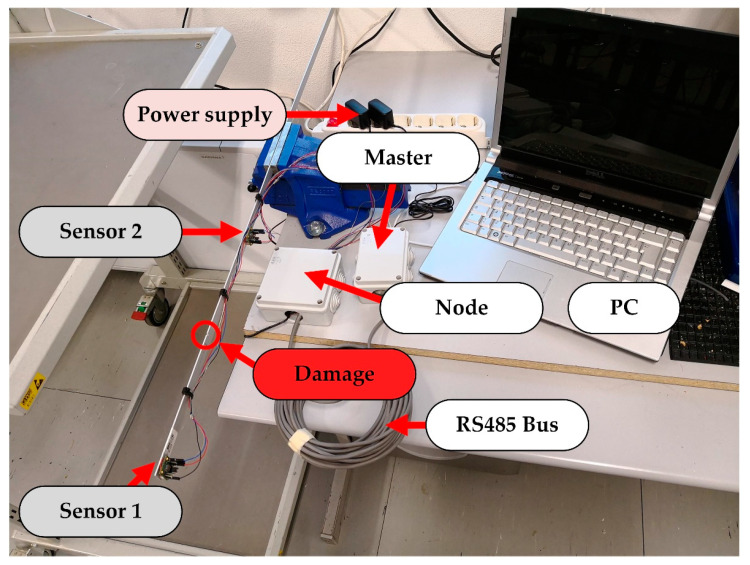
Experimental set-up of testing structure for the identification of the damage indicator.

For this test, one master and one node that communicate through the RS485 bus were used. The node acquires the data from two three-axis accelerometers, saves them on an SD card, and at the end of the test, transmits to the master device. Moreover, an external power supply for the node and master is required. A picture of the complete testing system is shown in Figure 4.

**Figure 5:**
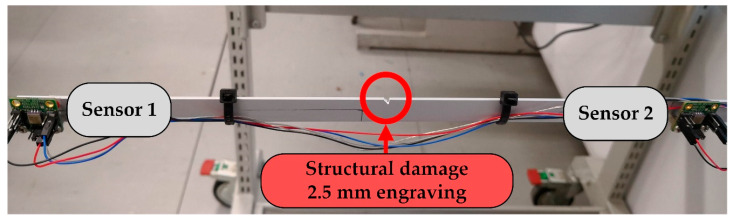
Engraved aluminum bar anchored in the bench vice for the damage detection test.

Having adapted the sampling frequency of 250 Hz, two tests have been carried out. The first test concerned the acquisition campaign with the healthy structure, and after that, the aluminum bar was damaged for the second experiment. For damage detection, a perturbation to the structure was induced. Indeed, on the testing structure (Figure 5), a 2.5 mm engrave was realized. For both the tests, the bar was stressed with only ambient noise. The approach of damage detection is based on an algorithm that processes the output data of the acquisition system when the structure is subjected to external excitations. These output data represent two measurements lasting 15 min of the healthy and damaged structure. The algorithm allows the evaluation of the damage indicator of a structure.

## 5. Results and Discussion

**Figure 6:**
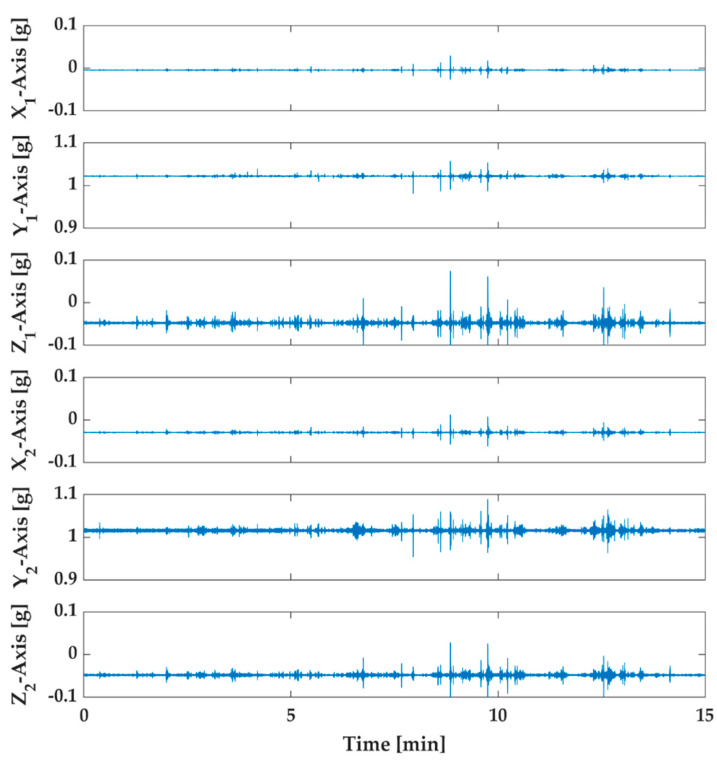
Six axes acquired data through the proposed monitoring system of the undamaged structure. The first three measurements are related to sensor 1 (X_1_, Y_1_, Z_1_), and the others are from sensor 2 (X_2_, Y_2_, Z_2_).

**Figure 7:**
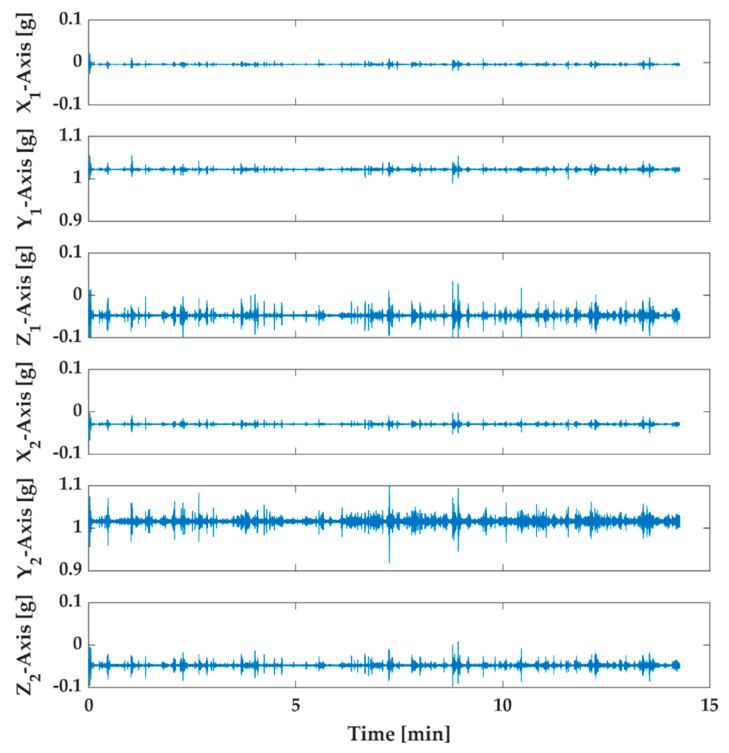
Six axes acquired data through the proposed monitoring system of the damaged structure (engraving of 2.5 mm). The first three measurements are related to sensor 1 (X_1_, Y_1_, Z_1_), and the others are from sensor 2 (X_2_, Y_2_, Z_2_).

Measurements have been performed on six axes, three for each triaxial sensor. In Figure 6 and Figure 7, the acceleration measurements relative to all axes located on the structure for the two tests are shown.

The acquired samples were 224,400 for the healthy structure and 213,602 for the damaged structure. Indeed, with sample time equal to 5 ms, the whole experimental time is about 15 min for the healthy structure and approximately 14.27 min for the damaged structure. In order to estimate the damage indicator, the first measurements have been divided into four series with about 50,000 samples each. These series have been called UD1, UD2, UD3, and UD0. The latter series UD0 has been used to calculate the damage indicator as the reference subspace. Similarly, the measurements of the damaged structure have been divided into four series called D1, D2, D3, and D4 with the same number of samples. 

**Figure 8:**
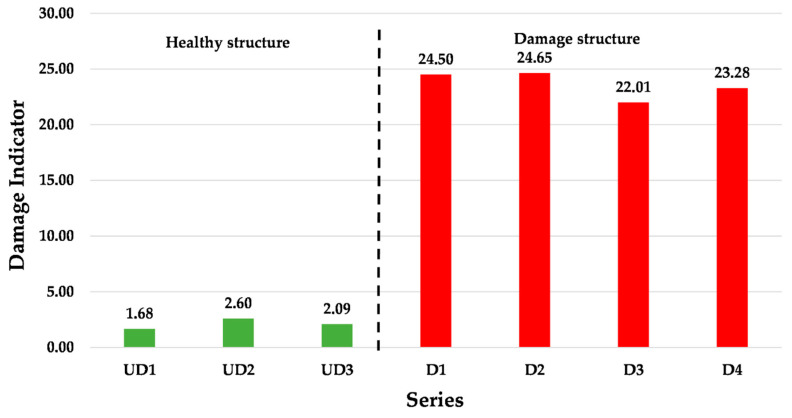
Values of the damage indicator. The green bars represent the structure in a healthy condition and the red bars show the damage indicator with the engraved structure.

Therefore, with the reference subspace UD0, the algorithm returns three damage indicator values for the healthy structure and four for the damaged structure. These values are smaller in structural health condition than the structural damage condition. The values of the damage indicator are shown in Figure 8.

Finally, in Table 1, the calculated damage indicators have been reported. The values of the damage indicator have an increase of ten times, with only a 2.5 mm engrave. The results show that the proposed system, with synchronous samples between the two sensors, is able to detect damages in a monitored structure. However, the proposed monitoring system with a damage indicator approach will detect structural defects or damage after events such as earthquakes or landslides.

## 6. Conclusions

In this work, an IoT monitoring system for structural health is presented. The IoT system, with application in Smart Buildings, allows for the measurement of the main parameters for evaluating the damage indicator. The system is based on the microcontroller Sam3X8E ARM cortex-M3 and high-resolution digital accelerometers ADXL355. Furthermore, thanks to the use of an SD card and DMA, the system allows for the acquisition of a high number of samples and communicates through the RS485 to the master device.

The reliable results have been ensured with the high synchronization between the sensors and their high resolution. Instead, the problems of the traditional analog sensors used in the typical monitoring systems have been eliminated with the use of the digital accelerometers. Therefore, the proposed monitoring system is cheaper than an analog solution.

The system has been used to evaluate damage in an aluminum bar locked in a bench vice. The test gave the possibility to assess, with the developed system, its capability of damage identification. An engraving was realized in the structure for comparing the evaluated damage indicator in both conditions.

Therefore, to implement a structural health monitoring system, the detection of any damage is important. Future development of the proposed system will concern the installation of the device on real structures, such as buildings.

## Figures and Tables

**Table 1:** Damage indicator values for all measurements in undamaged and damaged structures.

Condition	Series	Damage Indicator
Healthy Structure	UD1	1.68
	UD2	2.60
	UD3	2.09
Damaged Structure	D1	24.50
	D2	24.65
	D3	22.01
	D4	23.28
